# Medical image registration and its application in retinal images: a review

**DOI:** 10.1186/s42492-024-00173-8

**Published:** 2024-08-21

**Authors:** Qiushi Nie, Xiaoqing Zhang, Yan Hu, Mingdao Gong, Jiang Liu

**Affiliations:** 1 Research Institute of Trustworthy Autonomous Systems and Department of Computer Science and Engineering, Southern University of Science and Technology, Shenzhen, 518055 China; 2 Center for High Performance Computing and Shenzhen Key Laboratory of Intelligent Bioinformatics, Shenzhen Institute of Advanced Technology, Chinese Academy of Sciences, Shenzhen, 518055 China; 3 Singapore Eye Research Institute, Singapore, 169856 Singapore; 4 State Key Laboratory of Ophthalmology, Optometry and Visual Science, Eye Hospital, Wenzhou Medical University, Wenzhou, 325027 China

**Keywords:** Computer-aided diagnosis, Medical image registration, Deep learning, Generative model, Transformer, Retina

## Abstract

Medical image registration is vital for disease diagnosis and treatment with its ability to merge diverse information of images, which may be captured under different times, angles, or modalities. Although several surveys have reviewed the development of medical image registration, they have not systematically summarized the existing medical image registration methods. To this end, a comprehensive review of these methods is provided from traditional and deep-learning-based perspectives, aiming to help audiences quickly understand the development of medical image registration. In particular, we review recent advances in retinal image registration, which has not attracted much attention. In addition, current challenges in retinal image registration are discussed and insights and prospects for future research provided.

## Introduction

Medical image registration is a fundamental step in computer-aided diagnosis (CAD) and image-guided surgical treatment and has attracted much attention. It aligns multiple medical images by finding appropriate spatial transformation relationships to fuse their corresponding information, helping doctors make a more comprehensive and precise diagnostic conclusion. These medical images may be acquired at different times, angles, and even modalities for a certain tissue or organ of the human body. Therefore, the purpose of medical image registration is to eliminate the interference of these factors and find consistent objects or shapes for matching.

Numerous methods have been developed to address the different transformation tasks in medical image registration. These can be grouped into two types: coarse-grained global linear registration and fine-grained local elastic registration. Coarse-grained global linear registration extracts the salient features of the input image pair, thereby matching these features and overcoming angular changes. Fine-grained local elastic registration performs pixel-level analysis of the input image pair after linear alignment and local corrections to overcome spontaneous tissue movements and deformations.

Another method to classify registration methods is based on what is used to match the images. The first and direct approach is an intensity-based method [1]. These methods consider registration as an optimization problem by iteratively disturbing the transformation parameters to maximize pixel-wise similarity. Another early but still popular approach is feature-based methods [2], which extract manually designed features and descriptors, match them, and establish a transformation based on matching. In contrast to intensity-based methods, feature-based methods provide more robust registration by matching salient features rather than simply comparing pixels.

In the past decade, deep features have replaced handcraft features with their ability to provide learnable, and therefore, more flexible, problem-specific feature representations for registration tasks. Later, after deep feature extractors, end-to-end registration neural networks integrated the entire registration process into a single network by applying deep learning techniques such as convolutional neural networks (CNNs), generative adversarial networks (GANs), and transformers. Once trained, these methods can obtain registration results directly from input image pairs, thereby speeding up registration. They have also been proven to have better registration performance.

Several reviews on deep learning for medical image registration have been conducted [3–5]. However, those studies only investigated the popular CNN-based methods at the time and did not mention the latest transformer-based methods. Additionally, those studies only investigated methods based on deep learning but ignored traditional methods from the early years, which can also provide significant guidance.

Among medical images, retinal images focus on a unique part of the human body that allows noninvasive observation of blood vessels in vivo. This noninvasive approach allows the capture of high-quality images, which facilitates the examination of the retina with minimal discomfort to patients. Longitudinal studies critical for monitoring disease progression often use a series of images captured at various time intervals. The diagnosis of retinal diseases such as age-related macular degeneration (AMD) is further facilitated by the availability of multiple imaging modalities, each serving a distinct diagnostic purpose. AMD, characterized by choroidal neovascularization (CNV), exemplifies the need for a multimodal approach: (1) color fundus (CF) photography effectively highlights areas of hemorrhage and the presence of fibrovascular tissue; (2) fluorescein angiography (FA) reveals subtle leaks associated with CNV that are not always visible to the naked eye; and (3) optical coherence tomography (OCT) provides detailed cross-sectional scans that can uncover intraretinal abnormalities. These modalities collectively assist ophthalmologists in diagnosing retinopathies and formulating strategies for ophthalmic surgery [6]. Moreover, retinal analysis is relevant not only to eye diseases, but also to various human diseases, including diabetes [7], Alzheimer’s disease [8], and coronary heart disease [9]. Therefore, the retina serves as a microcosm for broader health assessments, providing a noninvasive yet informative window into a patient’s overall well-being. Retinal image registration, which combines complementary structural and functional information from the same or different modalities, is a crucial step in this process. Due to the particularity of the way retinal images are collected, they are mainly affected by three factors: illumination differences, angle differences, and variations in retinal lesions. These factors pose multiple technical challenges in the registration of retinal images: (1) Ensuring consistency in pixel values by standardizing or normalizing lighting conditions; (2) Identifying correspondences over long distances; (3) Tracking and quantifying the progression of retinal lesions.

However, in recent years, few studies have systematically reviewed retinal image registration. Although reviews have been conducted on related topics, such as retinal disease classification [10] and segmentation [11], the specific area of retinal image registration has not been thoroughly explored. Saha et al. [12], and Pan and Chen [13] addressed retinal image registration; however, they focused on a single retinal modality and did not perform a comparative analysis with mainstream medical image registration techniques. Therefore, the purpose of this paper is to review and summarize existing medical image registration works using traditional and deep learning-based methods, aiming to help audiences grasp the development of medical image registration. Moreover, retinal image registration are also surveyed and synthesized as a characteristic of this review. Finally, the current challenges in retinal image registration are also highlighted and future research directions discussed.

An initial literature search was performed using free-text searches in PubMed and Google Scholar. Papers that included the search term Medical Image Registration were considered and the publishing conference or journal and citations checked to ensure the quality of the research. Later, another search was performed using the search term Retinal Image Registration and all related papers considered. In the analysis, different temporal scopes were adopted for traditional and contemporary methods. For traditional methods the search was extended to encompass the last two decades, whereas for deep learning-based methods, the focus was narrowed to the most recent ten-year period to capture the latest advancements. Finally, the search space was iteratively increased by examining the bibliographies of the relevant papers.

**Fig. 1:**
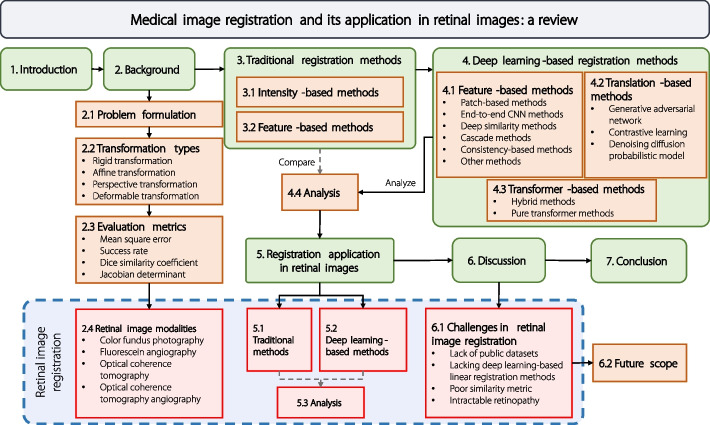
Structure of the review

The overall organization is illustrated in Fig. 1: Background section defines the basic concepts of image registration and briefly introduces the popular retinal image modalities. Traditional registration methods and Deep learning-based registration methods sections review the general methodology of medical image registration categorized as traditional and deep learning, respectively. Registration application in retinal images section reviews the applications in retinal image registration, and compares them with the general methodology. Discussion section discusses the advantages and disadvantages of the reviewed methods, highlights the current challenges, and provides potential future research directions. Finally, in Conclusion section, the paper is summarized.

## Background

### Problem formulation

Image registration is a fundamental task in image processing. This involves finding correspondences between two images, namely, a moving and fixed image, and establishing a transformation between them. A fixed image is used as a reference, and the goal is to transform the moving image to match the fixed image. Registration algorithms are designed to determine the best transformation, denoted by $T^*$, that maximizes the similarity between two images [14]. This can be achieved by maximizing the image similarity function $\text {sim}(I_f, T(I_m))$, where $I_m$ and $I_f$ are the moving and fixed images, respectively, and $T(I_m)$ is the moving image transformed using the transform *T*.

### Transformation types

**Fig. 2:**
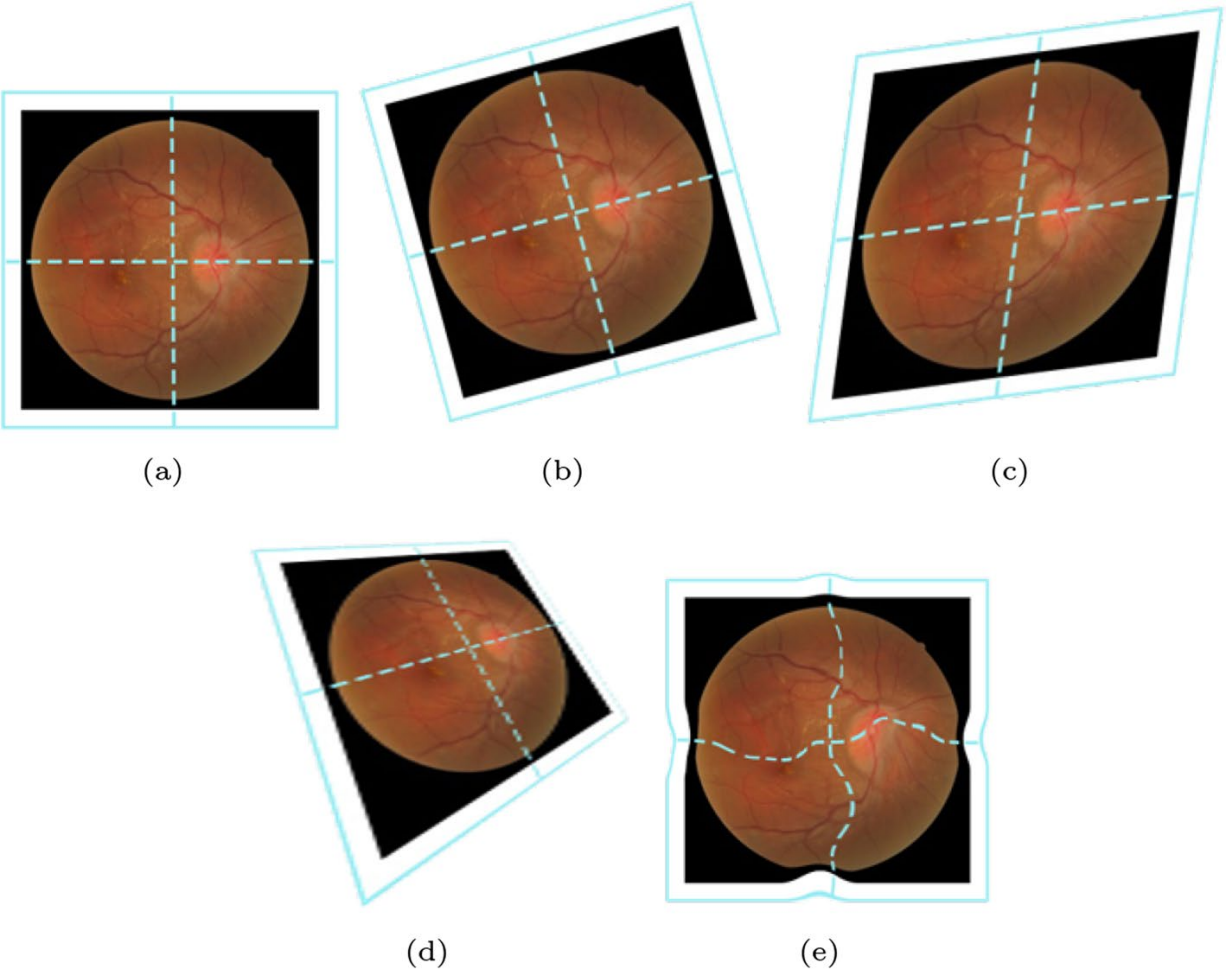
Effect of different transformations. **a** Origin; **b** Rigid; **c** Affine; **d** Perspective; **e** Deformable

This subsection introduces different transformation models, including rigid, affine, perspective, and deformable. Rigid, affine, and perspective transformations are linear, whereas deformable transformations are nonlinear. Figure 2 visually demonstrates their effects.

Rigid transformation consists of translation and rotation and preserves the original image’s size and shape. It is represented as:

1 $$\begin{aligned} \left[ \begin{array}{l} x'\\ y'\end{array}\right] = \varvec{R}\left[ \begin{array}{ll} x\\ y\end{array}\right] +\varvec{t} \end{aligned}$$

Here, (*x*, *y*) and $(x', y')$ denote the original and transformed pixel coordinates, $$\varvec{R}=\left[ \begin{array}{cc} \cos \theta & -\sin \theta \\ \sin \theta & \cos \theta \\ \end{array}\right]$$ is the rotation matrix, and $\varvec{t} = [t_x, t_y]^T$ is the translation vector.

Affine transformation combines translation, rotation, scaling, and shearing, offering more flexibility than rigid transformation. Affine transformation preserves straight lines and parallelism, but is not perpendicular. It is represented as:

2 $$\begin{aligned} \left[ \begin{array}{l} x'\\ y'\end{array}\right] = \left[ \begin{array}{cc} a & b\\ c & d \end{array}\right] \left[ \begin{array}{l}x\\ y\end{array}\right] + \varvec{t} \end{aligned}$$

Perspective transformation, or projective transformation, corrects perspective distortions, such as foreshortening and skew, between images. Perspective transformation maintains straightness but not parallelism or perpendicularity. This is represented in homogeneous coordinates as follows:

3 $$\begin{aligned} \left[ \begin{array}{l} x'\\ y'\\ w'\end{array}\right] = \left[ \begin{array}{lll} A & B & C\\ D & E & F\\ a & b & c \end{array}\right] \left[ \begin{array}{l} x\\ y\\ w\end{array}\right] \end{aligned}$$

Here, (*x*, *y*, *w*) is the homogeneous coordinate of the image to be transformed, $(x', y', w')$ is the target coordinate in the transformed image. By setting $w=1$ and transforming the target $w'=1$, the target point $(x', y')$ is obtained in Cartesian coordinates:

4 $$\begin{aligned} \begin{array}{l} x' = \frac{Ax+By+C}{ax+by+c}\\ y' = \frac{Dx+Ey+F}{ax+by+c}\\ \end{array} \end{aligned}$$

Deformable transformation allows nonlinear deformation, better adapting to shape variations compared to rigid or affine methods. It is represented as:

5 $$\begin{aligned} \left[ \begin{array}{l} x'\\ y' \end{array}\right] = \left[ \begin{array}{l} x\\ y \end{array}\right] + \phi [x,~ y] \end{aligned}$$

Here, $\phi$ represents the deformation field and $\phi [x, y]$ represents the transformation vector $(\Delta x,\Delta y)$ at (*x*, *y*).

### Evaluation metrics

Reliable evaluation metrics are crucial for assessing the medical image registration performance and guiding the design of new algorithms. Here, a brief review of four popular evaluation metrics is provided.

Mean square error (MSE) and root mean square error (RMSE) are standard metrics for measuring the quality of image registration. MSE can be calculated as

6 $$\begin{aligned} \text {MSE} = \frac{1}{N}\sum \limits _{i=1}^N(I-J)^2 \end{aligned}$$

RMSE simply adds an extra step to the square root based on MSE, and *I* and *J* have different meanings.

1. images: serves as image similarity measurement between warped moving image $I_M'$ and fixed image $I_F$.
2. point pairs: serves as the distance measurement between corresponding point pairs.
3. transformations: serves as the difference between ground truth transformation and predicted transformation.

Success rate (SR) quantifies the proportion of successful registrations out of the total number of registration samples. It can be mathematically expressed as

7 $$\begin{aligned} \text {SR} = \frac{N_{\text {success}}}{N_{\text {total}}} \times 100\% \end{aligned}$$

where $N_{\text {success}}$ is the number of successful registrations and $N_{\text {total}}$ is the total number of registration samples. However, the definition of *success* varies among studies, using different criteria or thresholds. The most commonly used criterion is the MSE between the predicted and corresponding ground truth points.

Dice similarity coefficient (DSC) quantifies the spatial overlap between two segmentations. For registration, Dice is calculated between the segmentation maps of the fixed image and the warped moving image to evaluate the overlap of the anatomical structures, which can be mathematically expressed as

8 $$\begin{aligned} \text {DSC} = 2\times \frac{|S_F\cap (S_M\circ \phi )|}{|S_F|+|S_M\circ \phi |} \end{aligned}$$

where $S_F$ and $S_M$ are the segmentations of $I_M$ and $I_F$, respectively, and $S_M\circ \phi$ represents the warped segmentation of the moving image using the transformation $\phi$.

Jacobian determinant quantifies the physical plausibility and invertibility of deformations by measuring how each pixel (or voxel if 3D) changes after the application of a certain deformation field. When the Jacobian determinant is non-positive, the deformation is not diffeomorphic. The percentage of pixels (or voxels) with non-positive Jacobian determinants ($|J_\phi |\le 0$) is always used, and the Jacobian determinant *J* at each point (*i*, *j*) of the deformation field $\phi$ can be formulated as

9 $$\begin{aligned} \text {det}(J_\phi (i, j)) = \left| \begin{array}{cc} \frac{\partial i}{\partial x} & \frac{\partial j}{\partial x} \\ \frac{\partial i}{\partial y} & \frac{\partial j}{\partial y} \end{array}\right| \end{aligned}$$

For 3D images, the 3D Jacobian determinant of each point (*i*, *j*, *k*) is used. This can be similarly defined as:

10 $$\begin{aligned} \text {det}(J_\phi (i, j, k)) = \left| \begin{array}{ccc} \frac{\partial i}{\partial x} & \frac{\partial j}{\partial x} & \frac{\partial k}{\partial x}\\ \frac{\partial i}{\partial y} & \frac{\partial j}{\partial y} & \frac{\partial k}{\partial y}\\ \frac{\partial i}{\partial z} & \frac{\partial j}{\partial z} & \frac{\partial k}{\partial z}\\ \end{array}\right| \end{aligned}$$

### Retinal image modalities

To illustrate retinal image registration, four commonly used techniques for photographing the eye are introduced: CF photography, FA, OCT, and optical coherence tomography angiography (OCTA). These techniques provide various medical imaging tools to analyze retinal conditions.

#### CF photography

**Fig. 3:**
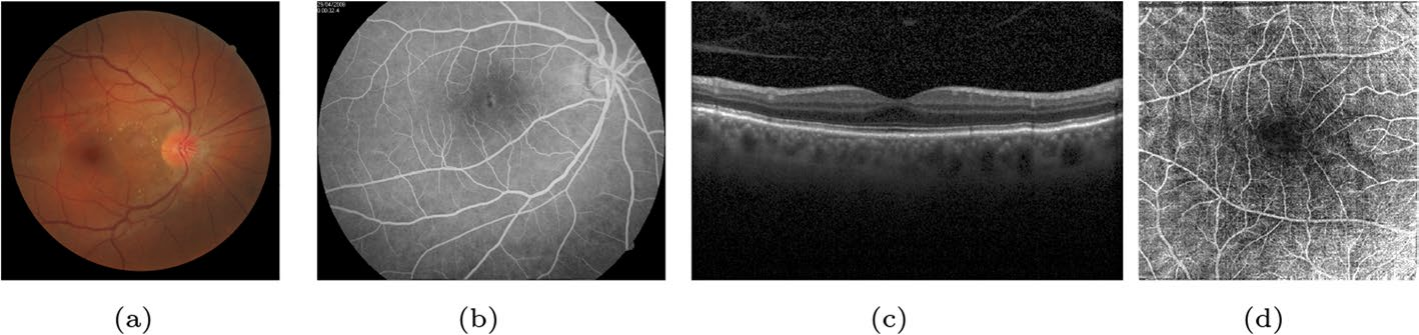
Fundus photography examples using different imaging techniques. **a** CF from FIRE dataset [24]; **b** FA from CF-FA dataset [25]; **c** OCT from ref. [26]; **d** OCTA from OCTA-500 dataset [27]

CF photography involves the use of a fundus camera to capture color images of the retina using white light. Equipped with a low-power microscope, the camera magnifies the interior surface of the eye. This technique is cost effective and straightforward for trained professionals [15]. The CF images (Fig. 3a) contain a broader range of fundus and rich color information, making it helpful in checking the atrophy of the retina and macular. Additionally, it helps diagnose retinopathies, such as diabetic retinopathy (DR), AMD, and glaucoma, as well as reveal signs of systemic diseases, such as diabetes and cardiovascular diseases [16].

#### FA

The FA, shown in Fig. 3b, involves a special dye called fluorescein and a camera to trace blood flow in the retina and choroid. It uses a special dye, fluorescein, and a camera to examine blood flow in the retina and choroid. The radiopaque dye is injected into the vein of the tester’s arm, and the retinal vessels are photographed by tracing the dye before and after injection. FA can be used to detect capillary leakage [17], aneurysms, and neovascularization. However, some people may experience discomfort after the procedure [18].

#### OCT

OCT is an imaging technology that uses the interference between an investigated object and a local reference signal to create high-resolution cross-sectional images and 3D scans of the retina and anterior segment [19]. Figure 3c shows a cross-sectional scan of OCT. It is a noninvasive technique that enables visualization of each layer of the retina, measurement of its thickness, and provides treatment guidance for conditions such as glaucoma, DR, and AMD. Intraoperative OCT (iOCT) is necessary in many retinal therapies, including glaucoma surgery [20] and epiretinal device implantation [21], because it provides real-time visualization of the retinal layers.

#### OCTA

Figure 3d showcases OCTA, an emerging imaging technology that builds upon OCT. OCTA captures images of the vascular network with a higher resolution and smaller view than FA without invasiveness. Using the decorrelation signal produced by moving blood cells, OCTA generates an image of the microvascular network. Recent studies have demonstrated the ability of OCTA to overcome the limitations of assessing blood flow in the optic nerve, explain the vascular pathogenesis of glaucoma [22] and show impressive success in preclinical DR diagnosis [23].

## Traditional registration methods

Researchers have developed increasingly sophisticated algorithms and resilient features during the initial image registration phase to achieve precise registration. This paper employs the phrase “traditional methods” to differentiate between the techniques utilized before the advent of deep learning and those implemented thereafter.

### Intensity-based methods

**Fig. 4:**
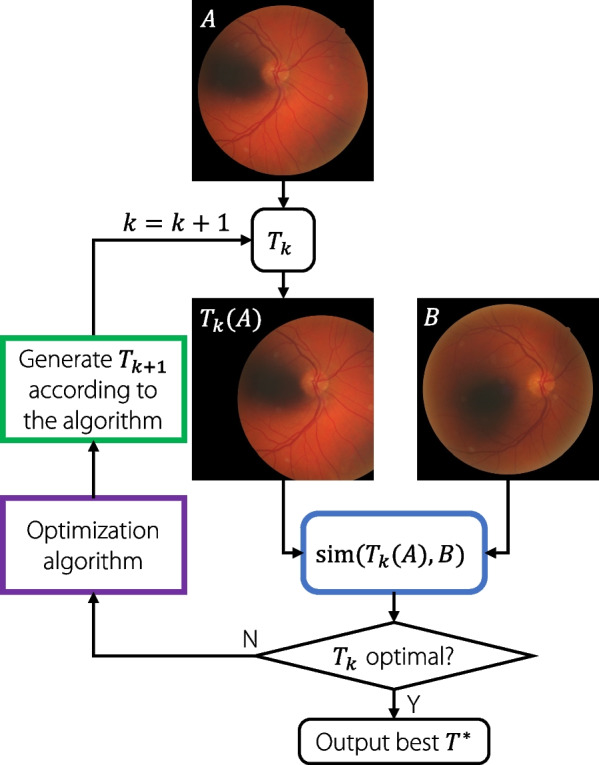
General registration procedure using iterative optimization

Intensity-based methods treat this problem as an iterative optimization problem. The basic steps of the intensity-based registration are shown in Fig. 4. Initially, a random transformation $T_0$ is selected, and an objective function is defined to measure the similarity between the transformed image $T_k(A)$ and another image *B*. The goal is to find the optimal transformation $T^*$ to maximize similarity. At each step, the optimization algorithm applies a perturbation to the parameters in *T* based on the current similarity measure $\text {sim}(T_k(A), T(b))$. The process is terminated when the similarity satisfies the requirement, or converges with no further increase.

Researchers have mainly concentrated on developing various similarity functions, including (normalized) cross-correlation (CC), (normalized) mutual information (MI), and sum of squared differences (SSD). These functions are typically calculated by using the difference between each corresponding pixel in an input image pair. MI is considered the most important and widely used function. The large-deformation diffeomorphic metric mapping [28] model is based on manifold learning theory and uses the Euler-Lagrange equation for optimization. It regards the image as a point on the manifold and achieves image registration by calculating the deformation between the manifolds. This model can handle large deformations and maintain the nonlinear structure of an image.

Recently, several studies have been conducted using intensity-based methods. Lange and Heldmann [29] proposed a normalized gradient field (NGF) distance measure for 2D-3D image registration. To overcome the drawback that standard similarity measures may lead to optimization problems with many local optima, Öfverstedt et al. [30] adopted a symmetric, intensity-interpolation-free similarity measure that combines intensity and spatial information. Castillo [31] proposed an intensity-based deformable image registration optimization formulation that is easier to optimize. The similarity function is designed as a simple quadratic function that can be solved using a straightforward coordinate descent iteration.

### Feature-based methods

**Fig. 5:**
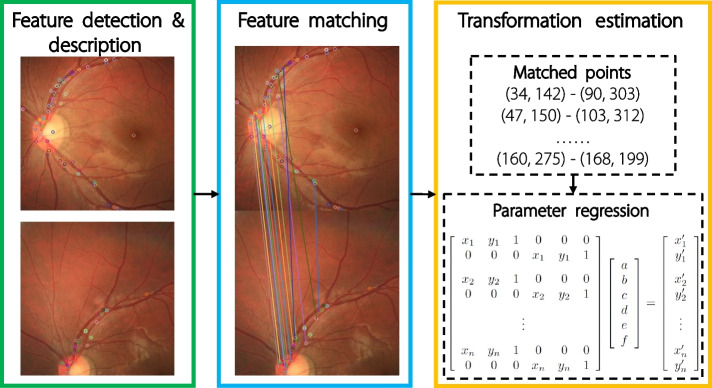
General keypoint-based registration procedure

Feature-based methods are popular methods of matching images based on their correspondence. These methods focus on the local structures and salient features of images, rather than on global information. The process is divided into three steps. First, features such as points, edges, and regions are extracted from the input images. Next, a descriptor is calculated for each feature. In the matching stage, the closest features of the two images are matched to establish potential correspondences. The idea is that the corresponding points should have similar descriptors. Finally, the transformation parameters are estimated based on the matching results. The primary challenge is to determine the most effective method for extracting and describing features. Figure 5 illustrates the keypoint-based registration process.

One pioneering work in feature-point-based registration is the scale-invariant feature transform (SIFT) [32]. SIFT transforms image data into scale-invariant coordinates, identifies stable keypoints, assigns orientations to keypoints, and generates feature descriptors for each keypoint. The extracted features are invariant under variations in scale, brightness, and angle. However, the process is computationally expensive. To address this problem, various efforts [33–37] have been made to enhance the performance and efficiency of SIFT. For instance, speeded up robust features (SURF) [33] simplify the filter function to reduce the dimensions of descriptors and improve computational efficiency. Another method, oriented FAST and rotated BRIEF (ORB) [36], integrates the FAST [38] keypoint detector and the BRIEF [39] descriptor to solve the high computational cost of SIFT features and the lack of rotation invariance, scale invariance, and sensitivity to noise in the BRIEF feature. As a result, ORB is capable of delivering a speedup of up to two significant figures compared with SIFT. Other studies have focused on edge and contour features using classic edge detection [40, 41] and image segmentation [42] algorithms for feature extraction.

## Deep learning-based registration methods

Deep learning-based image segmentation has proven to be a robust tool for image segmentation since 2019 [43]. These methods can improve accuracy and efficiency by automatically learning high-level features from the input images. Registration tasks, similar to segmentation, have been developed using deep learning methods. They differ from feature-based approaches because they utilize deep neural networks to replace feature extractors, feature matching, and transformation processes. Rather than directly optimizing the transformation parameters, these methods indirectly optimize the registration model parameters, thereby revealing the true essence of their effectiveness.

### Feature-based methods

The CNN is a pioneering work in computer vision. It uses learnable convolution kernels and inductive biases, such as locality and translation equivariance, to detect learned patterns in local regions and extract high-level features. This characteristic makes CNNs particularly suitable for object detection and image registration tasks, where spatial features are essential. Table 1 displays the prominent works on CNN-based registration methods, which have become the most popular approaches in the field since 2016.

**Table 1:** Overview of feature-based image registration methods

Reference	Year	Scene	Dimension	Modality	Type	TS	MM	Net architecture	Evaluation metric	Loss function
Miao et al. [44]	2016	Virtual	2D/3D	X-ray/CT	R	S	N	CNN regressor	SR/TRE	MSE
Yang et al. [45]	2017	Brain	3D	MR	D	S	N	Dual branch	MSE/$|J_\phi |$	MAE
Cao et al. [46]	2017	Brain	3D	MR	D	S	N	CNN regressor	Dice/ASSD	MSE
de Vos et al. [47]	2017	Digits/Heart	2D	Digit/MR	D	U	N	CNN regressor	Dice/95SD/ASSD	NCC
Zheng et al. [48]	2018	Bone	2D/3D	X-ray/CT	R	S	Y	Dual branch	RMSE/Error rate/TRE	MSE/PDA
Sloan et al. [49]	2018	Brain	3D	MR	R	S	N	CNN regressor	MAE	MSE
Chen and Wu [50]	2018	Brain	3D	MR	A	S	N	Siamese	Jacc/HD	MSE
Lv et al. [51]	2018	Abdominal	3D	MR	D	S	N	CNN regressor	SNR	NCC
Hu et al. [52]	2018	Prostate gland	3D	MR/US	D	W	Y	FCN	RMSE/Dice	Dice + Smooth
Jiang and Shackleford [53]	2018	Chest	3D	CT	D	U	N	CNN regressor	SSD	-
Li and Fan [54]	2017	Brain	3D	MR	D	U	N	FCN	Dice	NCC + Smooth
Fan et al. [55]	2019	Brain	3D	MR	D	S	N	FCN	Dice	MSE
Xu and Niethammer [56]	2019	Knee/Brain	3D	MR	D	W	N	FCN	Dice	Dice + NCC + Smooth
de Vos et al. [57]	2019	Heart/Chest	3D	MR/CT	A/D	U	N	Siamese	Dice/HD/ASSD/$|J_\phi |$	NCC + Smooth
Zhao et al. [58]	2019	Liver/Brain	3D	CT/MR	A/D	U	N	FCN	Jacc/Lm. Dist	CC + Smooth + Orthogonality + Determinant
Zhao et al. [59]	2019	Liver/Brain	3D	CT/MR	A/D	U	N	FCN	Dice/Lm. Dist	CC + Smooth
Balakrishnan et al. [60]	2019	Brain	3D	MR	D	W/U	N	FCN	Dice/$|J_\phi |$	MSE/CC + Dice + Smooth
Dalca et al. [61]	2019	Brain	3D	MR	D	U	N	FCN	Dice/$|J_\phi |$	Variational inference + Surface matching
Hu et al. [62]	2019	Brain	3D	MR	D	U	N	Siamese + Pyramid	Dice	NLCC + Smooth
Wang and Zhang [63]	2020	Synth./Brain	2D/3D	Eye/MR	D	S	N	Dual-FCN	Dice	MSE + Smooth
Mansilla et al. [64]	2020	Chest	2D	X-ray	D	W	N	FCN	Dice/HD/ASSD	NCC + Smooth
Mok and Chung [65]	2020	Brain	3D	MR	D	U	N	FCN	DSC/$|J_\phi |$	NCC + Pair + Smooth + Magnitude
Kim et al. [66]	2021	Face/Brain	2D/3D	Expr./MR	D	U	N	FCN	NMSE/SSIM/Dice/$|J_\phi |$	LCC + Smooth + Cycle + Identity
Czolbe et al. [67]	2021	Brain/Cell	3D	MR/EM	D	W/U	N	FCN	Dice	Cos sim of feature extractor
Mok and Chung [68]	2022	Brain	3D	MR	D	U	N	FCN	MAE/SR	NCC + Inverse + Smooth
Kang et al. [69]	2022	Brain	3D	MR	D	U	N	Siamese + Pyramid	Dice/ASSD/HD/$|J_\phi |$	NLCC + Smooth
Tran et al. [70]	2022	Liver/Brain	3D	CT/MRI	D	U	N	FCN	Dice/Jacc	Adversarial + Discrimination + Reconstruction
Kong et al. [71]	2023	Brain	2D/3D	CT/MR	D	U	Y	FCN	Dice/HD	Evaluator + Smooth
Che et al. [72]	2023	Brain	3D	MR	D	U	N	FCN	Dice/$|J_\phi |$/ASSD	NCC + Smooth + Anti-folding

For the **TS** (Training Strategy) column, S: Supervised, W: Weakly supervised, and U: Unsupervised. In the **MM** (multi-modal) column, Y: Yes, and N: No. In the **Type** column, R: Rigid, A: Affine, P: Perspective, and D: Deformable

**Fig. 6:**
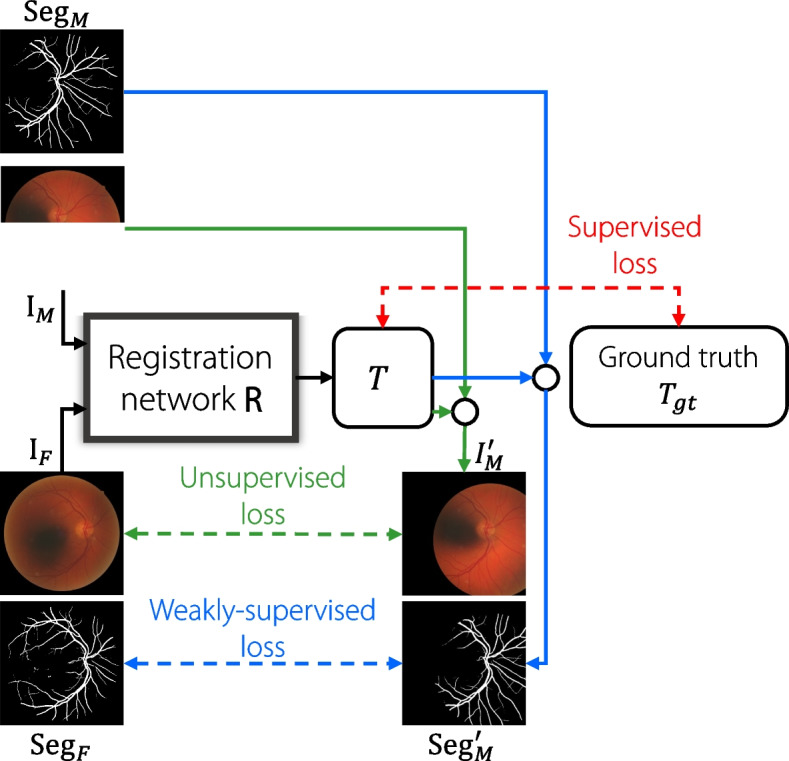
The overall framework for end-to-end deep learning-based medical image registration methods. The moving image $I_M$ and the fixed image $I_F$ are sent into the registration network *R*, and the output is obtained as the predicted transformation *T*. $\text {Seg}_M$ denotes the anatomical segmentation label of $I_M$ while $\text {Seg}_F$ denotes the anatomical segmentation label of $I_F$. The small circles denote performing transformation *T* on $I_M$ or $\text {Seg}_M$ using STN [75], gaining warped moving image $I_M'$ or warped label $\text {Seg}_M'$. Red, blue, and green lines denote the supervised, weakly-supervised, and unsupervised training strategies, respectively

#### Patch-based methods

Instead of the direct regression of registration parameters from the image pair, a patch-based approach is used to divide the image into smaller patches. The patch is utilized in different ways depending on the predicted transformation type. For linear transformations, the network establishes a match that can be used to derive the registration parameters. Conversely, a local displacement field is output and combined for nonlinear transformations. Various CNN models were proposed by Zagoruyko and Komodakis [73] that output the similarity between two image patches as feature descriptors. Cao et al. [46] proposed a similarity-steered CNN regression architecture that estimates the displacement vectors at each corresponding location between linearly aligned brain MR pairs. Interpolation is subsequently utilized to obtain a dense deformation. Lv et al. [51] divided the signal into three bins and used a CNN to estimate the displacement field for abdominal motion correction throughout the respiratory cycle. However, these methods typically require additional steps of patch selection and final registration, which can be time-consuming. In addition, the generation or manually labeling ground truth can be a limiting factor.

#### End-to-end CNN methods

Supervised end-to-end networks have been developed for direct registration owing to their increased computing power. The ground truth is obtained using traditional algorithms or manual labels. A general end-to-end deep learning registration framework is shown in Fig. 6. Miao et al. [44] employed 2D/3D CNN regressors to directly estimate rigid transformation parameters in real time. Quicksilver [45] divides 3D brain MRI into 3D patches owing to the limitations of GPU memory; however, it can directly predict the deformation field for the input patches. To improve the performance of supervised methods, Chee and Wu [50] leveraged unlabeled data to generate a synthetic dataset, and trained an affine image registration network. BIRNet [55] was proposed as a hierarchical dual-supervised fully CNNs based on U-Net [74] in the following year, with a loss function designed as a combination of the difference in image intensity and the difference in predicted displacement and ground truth displacement in each layer of U-Net’s decoder. Wang and Zhang [63] introduced a low-dimensional Fourier representation of diffeomorphic transformations to improve training and inference efficiency.

Weakly supervised registration methods take advantage of additional semantic information to ensure meaningful registration and overcome the challenge of the unavailability of ground truth transformations. These methods utilize additional information such as anatomical segmentation to perform registration. Hu et al. [52] proposed a weakly supervised registration network for multimodal 3D prostate gland images using the ground-truth segmentation labels of the gland and other anatomical landmarks. Xu and Niethammer [56] proposed a deep learning framework called DeepAtlas that jointly learns networks for image registration and segmentation, which are trained alternately and complement each other to achieve better results with only a few labels for segmentation.

Unsupervised methods have also been studied to eliminate the ground-truth labels. The spatial transformer layer (STL) [75], which is a differentiable module that can warp an input image, is the foundation of many unsupervised registration methods. STL enables the transformation of a moving image in a differentiable manner, allowing the application of conventional similarity measurements between the transformed and fixed images during training as the loss function. In 2017, DIRNet [47] was introduced as the first end-to-end unsupervised deformable registration network that adopted STL. Subsequently, VoxelMorph [60] was proposed as a U-Net-based network that achieved faster runtime and better performance than traditional iterative-based methods, with only unsupervised training. Auxiliary anatomical segmentation can be performed under weakly supervised settings. In their subsequent study, Dalca et al. [61] adopted a probabilistic generative model to provide diffeomorphic guarantees. Dual-PRNet [62] extended VoxelMorph [60] by incorporating a pyramid registration module that uses multilevel context information and sequentially warps convolutional features. Dual-PRNet$^{++}$ [69] further enhances the PR module in Dual-PRNet by computing the correlation features and using residual convolutions.

#### Deep similarity methods

Pixel-based similarity metrics, such as MSE and NCC, are commonly employed in deep learning. However, these metrics may encounter difficulties when dealing with low-intensity contrasts or noise. To address these issues, deep similarity methods that utilize custom similarity measures have been developed. For example, DeepSim [67] utilizes semantic information extracted by a pretrained feature extractor in a segmentation network to construct a semantic similarity metric. This specialized metric allows the network to learn and adapt to dataset-specific features, thereby improving the low-quality image performance. IMSE [71] takes this a step further with a self-supervised approach to train a modality-independent evaluator using a new data augmentation technique called shuffle remap, which can provide style enhancement. The evaluator then serves as a multimodal similarity estimator to train the multimodal registration network.

#### Cascade methods

Cascade methods were inspired by traditional iterative registration methods. The cascade architecture, that is, stacking networks in series, can provide progressive registration in a coarse-to-fine manner. DLIR [57] implemented a cascade architecture by stacking an affine network followed by multiple deformable networks, with each network being trained sequentially and the weights of the previous networks fixed. By contrast, Zhao et al. [58, 59] proposed a recursive cascade architecture similar to DLIR but much more sophisticated. They jointly trained their cascade networks to learn the progressive alignments more effectively.

#### Consistency-based methods

Consistency-based methods add consistency constraints based on the registration or transformation properties. In 2020, Mok and Chung [65] addressed the challenge of deformable transformation invertibility by introducing a swift and symmetric diffeomorphic image-registration approach. The network was trained with an inverse-consistency constraint, which enabled it to learn the bidirectional transformations of the mean shape of two input images to produce topology-preserving and inverse-consistent transformations. In the following year, Kim et al. [66] proposed CycleMorph, which utilizes cycle consistency as an additional constraint to enhance topology preservation and reduce folding issues. To register images X to Y and Y to X, the method employs two CNNs: $G_X$ and $G_Y$. The warped images from both networks are used as image pairs and sent to the networks themselves to ensure that they could be returned to their original state, maximizing the similarity between the original and reversed images.

#### Other methods

However, with the development of novel architectures, the number of parameters has increased significantly, making it more difficult to achieve real-time registration without high computing power. Tran et al. [70] attempted to solve this problem using knowledge distillation. They transferred meaningful knowledge of distilled deformations from a pretrained high-performance network (teacher network) to a fast, lightweight network (student network). After training, only a lightweight student network is used during the inference, allowing the model to achieve a fast inference time using only a common CPU.

### Translation-based methods

**Fig. 7:**
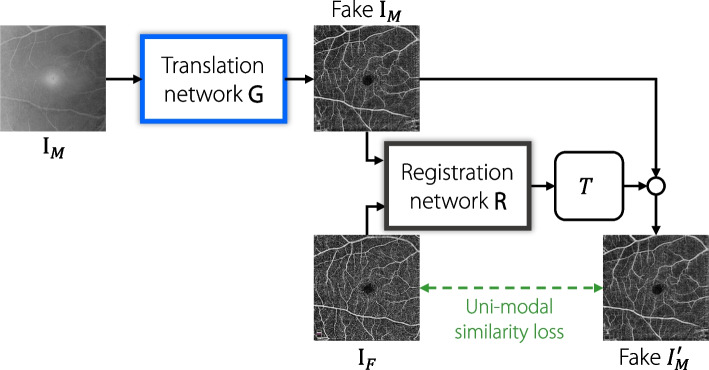
Overall framework for translation-based methods. The moving image $I_M$ is first sent into the translation network *G* which performs inter-modality translation and outputs the fake image $\text {Fake}\ I_M$. Then, $\text {Fake}\ I_M$ and the fixed image $I_F$ are sent into the registration network *R*, and the output is obtained as the predicted transformation *T*. The small circles denote performing transformation *T* on $\text {Fake}\ I_M$ using STN [75], gaining warped fake moving image $\text {Fake}\ I_M'$

Multimodal image registration can be complex, because it involves aligning images of varying modalities with unique intensity distributions. This poses a challenge for unimodal methods. However, an innovative solution to this issue is to leverage image translation techniques. This solution transforms the multimodal registration problem into a more straightforward unimodal registration problem, as shown in Fig. 7. Table 2 lists the most widely available translation-based registration algorithms.

**Table 2:** Overview of translation-based image registration methods

Methodology	Reference	Year	Scene	Dimension	Modality	Type	Evaluation metric
GAN	Mahapatra et al. [76]	2018	Retina/Heart	2D	CF/FA/MR	D	Dice/HD/ASD
	Qin et al. [77]	2019	Lung/Brain	2D	CT/MR	D	Dice/MCD/HD/RMSE
	Xu et al. [78]	2020	Kidney/Abdomen	3D	CT/MR	D	Dice/TRE
	Han et al. [79]	2022	Brain	3D	MR/CT	D	Dice/SD/HD/TRE
	Zhang et al. [80]	2023	Liver	3D	US	D	TRE
Contrastive learning	Casamitjana et al. [81]	2021	Brain	3D	Histology/MRI	D	RMSE/Dice
	Chen et al. [82]	2022	Thorax/Abdomen/Lung	3D	CT/MRI	D	Dice/HD95
DDPM	Kim et al. [83]	2022	Face/Brain	2D/3D	Expression/MR	D	Dice/$|J_\phi |$

For the **Type** column, R: Rigid, A: Affine, P: Perspective, and D: Deformable

#### GANs

A GAN [84] consists of two subnetworks, a generator and a discriminator, trained in a game-theoretic setting to generate synthetic data that are indistinguishable from the actual data. The generator generates synthetic samples, whereas the discriminator attempts to differentiate between natural and synthetic samples. The training process continues until the generated samples are indistinguishable from the actual ones.

Mahapatra et al. [76] used a GAN to generate a registered image with a distribution identical to that of the moving image and deformation field. They also ensured that the structure of the generated image matched that of the reference image through a structural similarity loss. Qin et al. [77] proposed a method for decomposing images into a latent shape space and separate latent appearance space for both modalities, which were used to learn a bidirectional registration function.

CycleGAN [85], which is based on GAN, enables image-to-image (i2i) translation using unpaired images. It employs cycle consistency loss to ensure that the reconstructed images are consistent with the original input images. Several multimodal registration methods [78, 79] have used CycleGAN as the primary network for image translation. Xu et al. [78] introduced two additional losses to enforce structural similarity between translated and authentic images. They also jointly trained the translated unimodal and multimodal streams to complement each other. Han et al. [79] implemented image synthesis in both directions and predicted the associated uncertainty, providing the information used in the fusion of the two direction estimations.

#### Contrastive learning

Contrastive learning defines positive and negative samples, and the goal is to learn a representation space where positive samples are close to each other and negative ones are far away. A recent study by Park et al. [86] explored the integration of contrastive learning into image translation by introducing an additional loss called patchNCE to a naive GAN. This loss encourages the generated output patches to be closer to their corresponding image patches than to random ones. Casamitjana et al. [81] used patchNCE loss to train an i2i translation network for transferring source images to the desired target domain. Subsequently, they applied an independently trained intramodality registration network to the target domain to predict the deformation field. Building on this work, Chen et al. [82] proposed an end-to-end architecture that jointly trains registration and translation networks without requiring a discriminator.

#### Denoising diffusion probabilistic model

A new generative model called the denoising diffusion probabilistic model (DDPM) [87] was recently introduced. This model is designed to learn Markov transformation from a simple Gaussian distribution to an actual data distribution. DDPM has been shown to generate images of higher quality than GAN [88]. In addition, Kim et al. [83] developed DiffuseMorph, which is the first and currently the only registration network based on diffusion. The network estimates the score function by adding a diffusion network before a standard registration network, and even shows the image registration trajectory by scaling the conditional score. However, unlike translation between modalities, DiffuseMorph constructs a score function directly between the input image pairs.

### Transformer-based methods

Recently, Google explored a method to use a pure transformer architecture in vision tasks, known as a vision transformer (ViT) [89], achieving competitive performance compared to existing CNN methods. ViTs split the image into patches and treat them as tokens, as in an NLP application, which has led to their successful application in various computer vision tasks, including image registration. Table 3 presents transformer-based image registration methods.

**Table 3:** Overview of transformer-based image registration methods

Reference	Year	Scene	Dimension	Modality	Type	Net architecture	Evaluation metric	Loss function
Chen et al. [90]	2021	Brain	3D	MR	D	Hybrid	Dice	MSE + Smooth
Zhang et al. [91]	2021	Brain	3D	MR	D	Hybrid	-	-
Mok and Chung [92]	2022	Brain	3D	MR	A	Pure	Dice/HD	NCC + Dice
Chen et al. [93]	2022	Brain/Heart	3D	MRI/XCAT/CT	A/D	Hybrid	Dice/$|J_\phi |$/ SSIM/HD	(MSE/LNCC) + Dice + Smooth
Song et al. [94]	2022	Brain	3D	MR	D	Hybrid	Dice/$|J_\phi |$	(MSE/LNCC) + Smooth
Wang et al. [95]	2022	Brain	3D	MR	D	Hybrid	Dice	LCC + Smooth
Shi et al. [96]	2022	Heart	3D	CT	D	Pure	Dice/$|J_\phi |$	Sim + Smooth
Zhu and Lu [97]	2022	Brain	3D	MR	D	Pure	Dice/$|J_\phi |$	MSE + Smooth + Determinant + Inverse
Chen et al. [98]	2023	Brain	3D	MR	D	Hybrid	Dice/HD/$|J_\phi |$	(LNCC/MSE) + Smooth
Wang et al. [99]	2023	Brain	3D	MR	D	Hybrid	Dice/ASSD/$|J_\phi |$	NCC + Smooth

For the **Type** column, R: Rigid, A: Affine, P: Perspective, and D: Deformable

#### Hybrid methods

Initially, researchers attempted to integrate Transformers into CNN-based models. Chen et al. [90] pioneered the use of ViT on high-level features extracted from the convolutional layers of moving and fixed images. Building on this approach, Song et al. [94] proposed TD-Net, which utilizes multiple transformer blocks for downsampling. Conversely, Zhang et al. [91] introduced a dual transformer network comprising two branches, intra-image and inter-image, with transformers embedded in both branches to enhance the features, similar to the approach in ref [90]. Wang et al. [95] enhanced the UNet [74] architecture for registration by introducing a bilevel connection and a unique transformer block. TransMorph [93] was proposed as a hybrid transformer-ConvNet model that utilizes Swin transformers [100] in the encoder and convolutional layers in the decoder. The authors demonstrated that positional embedding can be disregarded, leading to a flatter loss landscape for registration. The following year, Chen et al. [98] proposed TransMatch, emphasizing the importance of inter-image feature matching. They employed a transformer-based encoder and matched the regions using their new local window cross-attention module. Recently, Wang et al. [99] introduced a motion decomposition transformer based on a multihead neighborhood attention mechanism that can model multiple motion modalities.

#### Pure transformer methods

An alternative method involves the integration of a pure transformer architecture into a network. In a recent study by Shi et al. [96], a unique X-shaped transformer architecture called XMorpher was introduced. The researchers incorporated cross-attention between two feature extraction branches and a window-size constraint to enhance the information exchange and locality of the network. In another study, Swin-VoxelMorph [97] utilized a fully Swin transformer-based 3D Swin-UNet and a bidirectional constraint to optimize both forward and inverse transformations. To fill this gap in affine image registration, Mok and Chung [92] proposed a Coarse-to-Fine vision transformer, a pure transformer architecture. The researchers transformed the image pairs into small-to-large resolutions and passed them through different stages of ViT to achieve the desired results.

### Analysis

The evolution of image registration methods has been closely tied to advancements in computing power and deep-learning architectures. In the early stages, when computing power was limited, patch-based methods predominated. However, as computational capabilities and network diversity have expanded, it has become feasible to process entire images, and even 3D volumes, in a holistic manner. This shift facilitated the simultaneous and integrated performance of feature extraction and matching tasks. Concurrently, the feature extraction component of image registration has been progressively enhanced by the rapid development of deep-learning architectures.

Translation-based methods are effective in mitigating multimodal registration challenges by aligning image pairs within the same modality, thereby simplifying the registration process. Recently, there has been a surge in generative network-assisted registration methods that capitalize on the latest advancements in generative network models. Although GANs have shown promise in modality translation, their training process is notably complex and demands meticulous manual hyperparameter tuning for both the generator and discriminator components. Previously, contrastive learning dominated the unsupervised learning landscape; however, it requires extensive high-quality datasets for effective training. Diffusion models have recently emerged as promising image-generation techniques capable of producing highly realistic effects. However, its potential application in image registration remains an open research area.

In a CNN, the convolution operations are typically localized, focusing on extracting features from within a specific neighborhood. By contrast, the transformer architecture, with its self-attention mechanism, offers a distinct advantage by facilitating the exchange of information across the entire image. This capability is a key factor driving the integration of transformer models into registration networks because it significantly enhances the feature extraction process by considering global contextual information. There is also a trend towards the development of pure transformer architectures that have exhibited remarkable performances in various visual tasks. However, adapting the attention mechanism to suit specific requirements of image registration remains a problem. Therefore, cross-attention transformers are being investigated for their potential to refine the feature extraction phase and improve the feature-matching stage. This tailored approach can lead to more effective and robust registration methods, particularly for complex multimodal imaging scenarios.

By shifting the focus to the architecture of neural networks, distinct preferences in medical-image registration were observed. For linear registration, the CNN regressor stands out as the favored architecture owing to its versatility in both feature extraction and direct regression for obtaining linear registration parameters. By contrast, fully convolutional networks (FCN), particularly those resembling the UNet architecture [74], are favored for nonlinear registration. This is because of the FCN’s ability to produce a deformation field that corresponds to the size of the input image, making it exceptionally suitable for such tasks. The FCN architecture typically includes an encoder-decoder framework, with the encoder responsible for feature extraction and the decoder responsible for analyzing these features to generate results. A skip connection between the encoder and decoder facilitates the integration of the extracted features, enhancing the predictive capabilities of the network. Interestingly, more recent transformer-based models, which have had a significant impact, often adhere to this fundamental structure.

Building upon the FCN, derivative models such as the Siamese network and dual-branch network have been developed. These models employ two encoders that independently extract features from the input image and subsequently interact with and merge these features. In the context of single-modal registration tasks, the Siamese network, which shares weights between two encoders, is commonly utilized for its efficiency. However, in multimodal registration tasks, this approach diverges by employing two distinct encoders with separate weights to adaptively extract consistent features from two different modalities. Furthermore, to achieve a more robust deformation field, certain networks have been designed to output a pyramid of multiscale deformation fields. These fields are then integrated to form the final deformation field. The advantage of this multiscale approach is that it incorporates features from various levels of detail, rather than relying solely on the features produced by the decoder’s final output.

Innovation in loss functions is a critical aspect in the development of neural networks for medical image registration. In supervised training, the MSE between the predicted and true transformation parameters is the prevalent choice for the loss function. This metric provides a straightforward quantification of prediction accuracy. When shifting to unsupervised training, in which ground-truth transformation parameters are unavailable, image-similarity measures become essential. The most widely utilized image similarity losses include the MSE and CC. The CC, in particular, is calculated as follows:

11 $$\begin{aligned} \text {CC}(X, Y) = \frac{\text {Cov}(X, Y)}{\sqrt{\text {Cov}(X, X)\text {Cov}(Y, Y)}} \end{aligned}$$

where $\text {Cov}(X, Y)=\frac{1}{|\Omega |}\sum _{x\in \Omega }X(x)Y(x)-\frac{1}{|\Omega |^2}\sum _{x\in \Omega }X(x)\sum _{y\in \Omega }Y(y)$ is the covariance. In weakly supervised training scenarios, an additional loss function often comes into play-the dice loss, which is predicated on the segmentation labels of image pairs. This loss function is particularly adept at capturing spatial agreement between segmentations. Moreover, for nonlinear registration tasks, it is crucial to incorporate a smoothing penalty term into the loss function. This term encourages smoothness in the deformation field by promoting similarity in deformation quantities across adjacent positions. The most favored penalty term is the diffusion regularizer, which is mathematically expressed as:

12 $$\begin{aligned} R_{\text {diff}}(\phi ) = \sum ||\nabla _\phi ||^2 \end{aligned}$$

Furthermore, the incorporation of novel constraints that leverage the fundamental properties of the registration and transformation processes results in the creation of more refined output transformations. This approach is the cornerstone of consistency-based methods, which aim to ensure that the transformations generated by the network closely adhere to the underlying physical and geometrical principles of the registration task. In addition to these advancements, deep similarity methods have introduced the concept of training an evaluator or a custom similarity function to serve as a network’s loss function. This approach enables the network to automatically learn an appropriate similarity metric that aligns with the specific characteristics and requirements of the task.

While traditional methods require cumbersome iterative optimization calculations, which result in significant time consumption, deep learning-based approaches offer a notable efficiency advantage by allowing data to be input into the network during testing and providing results immediately after training. Furthermore, from a preprocessing standpoint, both traditional and deep learning-based methods require the downsampling of typically collected high-resolution medical images [101]. Utilizing the original scale would not only amplify the search space for the iterative optimization algorithm but also increase the number of parameters required by the deep learning network, imposing substantial overhead on both methodologies. Nevertheless, traditional methods have the advantage of delivering more stable outcomes and are convenient for plug-and-play applications. By contrast, deep learning-based methods require specialized training for each task. The trained model becomes obsolete when the application context shifts.

## Registration application in retinal images

### Traditional methods

First, intensity-based methods for retinal image registration were explored. The aforementioned intensity similarity metrics, such as MI [102–104] and CC [105], were used. Feature-based methods are more effective than intensity-based methods for retinal image registration. One popular approach is to use typical landmarks in retinal images. In 2003, Stewart et al. [106] introduced a Dual-Bootstrap Iterative Closest Point (Dual-Bootstrap ICP) algorithm for retinal image registration. This algorithm begins by matching individual vascular landmarks and aligning images based on the detected blood vessel centerlines. Other studies have utilized vascular features [107–110] and optical discs [111] for registration purposes.

One potential solution is to enhance the capabilities of keypoint detectors and feature descriptors to improve their performance. Ramli et al. [112] designed a D-sadle detector capable of detecting feature points even in low-quality regions. Yang et al. [113] built upon previous work [106] to create the generalized dual-bootstrap iterative closest point, which uses better initialization, robust estimation, and strict decision criteria to align retinal images from different modalities. Chen et al. [114] implemented a Harris detector to identify corner points, extract partial intensity-invariant feature descriptors, and perform bilateral matching between image pairs. The outliers are then removed, and the final transformation is applied. Ramli et al. [112] improved the saddle detector to detect feature points in low-quality regions. Gharabaghi et al. [115] utilized affine moment invariants as shape descriptors. By combining the domain knowledge, SIFT and its variants are used in refs [116, 117]. Li et al. [118] introduced orientation-independent feature matching that uses a new circular neighborhood-based feature descriptor.

### Deep learning-based methods

In this subsection, we review deep learning-based methods for retinal applications, categorized as outlined in “Deep learning-based registration methods” subsection. Table 4 summarizes the deep learning-based retinal image registration methods.

**Table 4:** Overview of deep learning-based retinal image registration methods

Reference	Year	Modality	Type	TS	MM	Net architecture	Evaluation metric	Loss function
Lee et al. [119]	2019	CF/FA /OCT	A	U	Y	CNN regressor	SR/RMSE/MAE/MEE	-
Zhang et al. [120]	2019	CF/FA	D	W	Y	FCN	Dice	Style + Content + MSE + SSIM + Smooth
De Silva et al. [121]	2020	CF/F/IR	A/D	S	Y	Siamese + CNN regressor	Reg. error	Overlap + Displacement
Wang et al. [122]	2020	CF/IR	P	S	Y	Seg. + Det. and Desc. + Out. Rej.	SR/Dice	CE + MSE + Dice
Tian et al. [123]	2020	CF/OCT	D	U	Y	FCN + Pyramid	MSE/HD/MSSIM	CC + Edge + Smooth
Zou et al. [124]	2020	CF	D	U	N	FCN + Pyramid	PA/Dice/RMSE	NCC + Smooth
Wang et al. [125]	2021	CF/FA/IR	P	S	Y	Seg. + Det. and Desc. + Out. Rej.	SR/Dice	CE + MSE + Dice
Zhang et al. [126]	2021	CF/FA/IR	A/D	S	Y	Seg. + Det. and Desc. + Out. Rej. / FCN	Dice	CE + MSE + Dice/Style + MSE
Sui et al. [127]	2021	MSI	D	W	N	FCN + Pyramid	TRE/Dice	Sim + Smooth
An et al. [128]	2022	CF/FA/IR	R	U	Y	Seg. + Det. and Desc. + Out. Rej.	SR/Dice	Pos. + Desc. + Score + CE + (MSE/Dice)
Benvenuto et al. [129]	2022	CF	D	U	N	FCN	MSE/SSIM/Dice	NCC
López-Varela et al. [130]	2022	OCTA	D	U	N	FCN + Pyramid	MSE/NRMSE/SSIM/VIF	LNCC
Rivas-Villar et al. [131]	2022	CF	S	S	N	FCN	RMSE/AUC	MSE
Kim et al. [132]	2022	CF	P	S	N	FCN	AUC	CE + Focal + Smooth
Santarossa et al. [133]	2022	CF/FAF/FAG	P	S	Y	CNN regressor	AUC	Relaxed Ranking
Liu et al. [134]	2022	CF	P	S	N	Det. and Desc.	EER	Det. + Desc.
Rivas-Villar et al. [135]	2023	OCT	A+Z	U	N	Det. and Desc.	Error	Repeatability + Reliable
Liu and Li [136]	2023	CF	P	U	N	CNN + Attn.	AUC/SR	CE

For the **TS** (Training Strategy) column, S: Supervised, W: Weakly supervised, U: Unsupervised. For the **MM** (multi-modal) column, Y: Yes, N: No. For the **Type** column, R: Rigid, S: Similarity, A: Affine, P: Perspective, Z: Z-axis, and D: Deformable

#### Feature-based methods

The identification of retinal landmarks has been a catalyst for the development of deep learning techniques. In particular, ref. [119] used handcrafted features, whereas [131, 132] used CNNs. Specifically, Lee et al. [119] employed a CNN to classify patches of various step patterns based on intensity changes. By contrast, Rivas-Villar et al. [131] used a CNN to produce a heatmap of blood vessels and bifurcations, and applied the maximum detection and feature matching method RANSAC [137] during testing. Similarly, Kim et al. [132] used a vessel segmentation network and joint detection network to identify vascular landmark points for registration. The SIFT algorithm [32] is then used to compute the descriptors based on the regions around these points. Benvenuto et al. [129] used an Isotropic Undecimated Wavelet Transform to segment blood vessels and ocular shapes. Based on the segmentation, the registration network adopted from U-Net is trained to perform registration. This year, Rivas-Villar et al. [135] explored deep learning registration methods for OCT 3D Scan. They first performed affine alignment on a 2D projection, followed by z-axis registration based on layer segmentation.

Recent studies explored the potential of end-to-end methods that utilize innovative network architectures. De Silva et al. [121] developed a model that employs a VGG 16 feature extractor, a correlation matrix, and a regression network to emulate the traditional feature-based registration pipeline, encompassing feature extraction, matching, and computation of the registration transformation; the effectiveness of their model was evaluated on a multimodal retinal dataset. Tian et al. [123] enhanced the U-Net architecture [74] by incorporating an image pyramid for multiscale input and introduced a novel edge similarity loss calculated through the correlation between the gradients of the fixed and moving images. However, based on U-Net, Sui et al. [127] further refined this approach by feeding an image pyramid of the original image and a ground truth vessel map into each layer of the encoder and decoder, respectively. Liu et al. [134] proposed SuperRetina, an end-to-end method with jointly trainable keypoint detector and descriptor.

It is worth noting that Wang et al. [120, 122, 125, 126, 128] made significant contributions to multimodal retinal image registration. They initiated their work using a deformable registration model comprising a vessel segmentation network and a deformation field estimation network, as described in ref. [120]. In their subsequent study [122], they refined the vessel segmentation network from their prior work and integrated a pretrained superpoint model [138] for feature detection and description, complemented by an outlier rejection network to facilitate perspective registration. This three-stage methodology, consisting of segmentation, detection, description, and outlier rejection, was subsequently employed in ongoing research. A notable advantage of this approach is its ability to bridge the intensity gap between different modalities; however, the complexity of the methodology remains a drawback. They further improved the segmentation network using pixel-adaptive convolution [125]. In ref. [126], the authors introduced perspective registration as a coarse step, followed by the addition of a deformable framework for fine alignment to achieve remarkable accuracy. Most recently, ref. [128] transformed the three-stage approach into a self-supervised process.

#### Translation-based methods

Although numerous studies have been conducted on i2i translation in various retinal modalities [139, 140], few studies on retinal image registration have used translation-based techniques. MedRegNet [133], which utilizes CycleGAN [85] as an image-translation tool, is the only available method of its kind. However, it is primarily employed as a generator of multimodal retinal data, rather than as a registration tool. The aforementioned work [120, 122, 125, 126, 128] can also be regarded as translation-based when addressing multimodal data. These studies capitalize on image segmentation to produce blood vessel segmentation maps, effectively converting different modalities into a unified ‘mask’ modality for registration purposes.

#### Transformer-based methods

Research on transformer-based retinal image registration methods is still in its infancy. GeoFormer [136] is the first method to adopt an advanced transformer-based attention blocks for detector-free feature matching on retinal images. It enhances coarse features by using geometrically matched regions rather than entire images, resulting in more accurate coarse matches.

### Analysis

In the domain of retinal image registration, traditional approaches have extensive applications and often employ various retinal modalities. Some studies have integrated domain-specific knowledge into general registration methodologies. However, these intensity-based methods can be sensitive to variations in illumination across image pairs, which may arise from differences in camera settings, imaging modalities, or changes in the retinal background due to retinopathy. This sensitivity is a common challenge affecting feature-based methods that require robust feature descriptors to perform well. Moreover, a significant drawback of many conventional registration techniques is their long inference times.

Deep learning-based methods for retinal image registration emerged more recently in 2019 and can be categorized into two main approaches. The first approach leverages state-of-the-art network architectures within the framework of mainstream registration methods. Although these methods deliver exceptional performance, their reliance on architectural design for domain-specific insights is notable. By contrast, the second approach aims to address the registration challenge in a manner that is more tailored to the domain. This involves extracting or utilizing key features such as vessel segmentation or vascular junctions for subsequent registration processes.

It has been observed that the diversity of approaches in retinal image registration appeared to be considerably lower than that in other areas of medical image registration. This can be attributed to several factors, including differences in the imaging principles and targets. For instance, imaging modalities such as CT and MR utilize X-rays and magnetic fields to generate images with high tissue contrast while maintaining consistent intensities across various acquisitions. By contrast, retinal image registration commonly relies on CF and FA images, which depend solely on white light illumination. The unique imaging principle of retinal imaging, combined with the natural movement of the subject’s eyeballs, can result in significant brightness variations within a sequence of images. Furthermore, the imaging target itself differs, while CT and MR are often used to image areas rich in features, such as the chest, abdomen, and brain. Retinal images predominantly focus on the vasculature and the optic disc, which exhibit less distinctive features. Consequently, learning the robust features for retinal image registration using deep learning is inherently challenging.

This survey revealed the scarcity of translation- or transformer-based approaches within the domain of retinal image registration. Notably, the majority of transformer-based studies have been conducted using MRI datasets. This trend can be attributed to the increased availability of public MRI datasets, which offer a wealth of data for research purposes. In addition, the ViT model, a prominent example of a transformer-based architecture, requires a substantially larger dataset to surpass the performance of conventional CNN models. While strategies such as data augmentation and the employment of pretrained models may offer provisional relief to this challenge, the crux of the solution lies in more publicly available data.

## Discussion

### Challenges in retinal image registration

#### Lack of public datasets

In artificial intelligence, many tasks rely on competition and public evaluations to make progress. These challenges offer a comprehensive and impartial platform for researchers to compare the performance, computation time, and robustness of newly designed algorithms. The Learn2Reg challenge, for example, recently focused on registering medical imaging modalities commonly used in the brain, abdomen, and thorax [141]. The datasets currently available for retinal image registration are listed in Table 5. Sufficient public retinal image datasets have not been formed for each modality, nor has there been competition.

**Table 5:** Public retinal image registration datasets

Dataset	Source	Camera specifications	Format	Modality	Resolution	Size (pairs)	Ground truth
FIRE [24]	Papageorgiou Hospital, Aristotle University of Thessaloniki, Greece	Nidek AFC-210 fundus camera	JPG	CF	2912$\times$2912	134	Control points
FLoRI21 [142]	RECOVERY study [143]	Optos California and 200Tx cameras	TIFF	UWF FA	3900$\times$3072	15	Control points
CF-FFA [25]	Unknown	Unknown	JPG	CF & FFA	720$\times$576	60	None

#### Different transformation type used from mainstream medical image registration

**Fig. 8:**
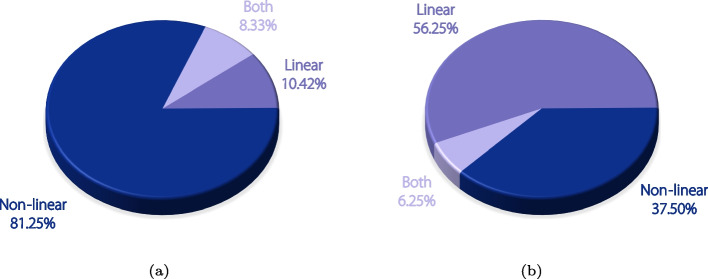
Comparative analysis of deep learning-based method using different transformation types. Pie chart (**a**) illustrates the distribution of different transformation types in general medical image registration, while pie chart (**b**) displays the distribution in retinal image registration

Based on articles using deep learning, the proportion of different transformation types used in the general medical image registration and the proportion of each type, specifically in the retinal image registration were calculated, as shown in Fig. 8. It was found that over 80% of studies on general medical image registration employed nonlinear transformations. On the contrary, linear transformation is the most commonly used method in retinal applications. This is because retinal images are primarily captured from a limited area of the retina while other commonly used modalities are 3D images with the subject completely contained in the image. Such difference in transformation types makes it difficult for retinal image registration to learn from mainstream medical image registration.

#### Poor similarity metric

Similarity metrics are used to optimize the registration network in an unsupervised manner or to evaluate the quality of the registration. The key technical challenge in medical-image registration is the selection and design of the most effective similarity measurement methods. Brightness changes may be the most significant difficulty in unimodal image registration. One of the main obstacles to multimodal image registration is that images from different modalities have different resolutions, contrasts, and luminosities. Therefore, a newly designed similarity metric, or a completely different technical route for multimodal image registration, is urgently required.

#### Intractable retinopathy

During clinical treatments, most patients experience eye retinopathy; therefore, their retinas may be severely damaged. Small bulges, swellings, or blood may cover the normal fundus and negatively affect photography. Some diseases alter the retinal structure. Most samples in the public datasets are retinal images from ordinary people. However, when used for clinical diagnosis, the retinas of some patients are likely to have retinopathy. In this case, a network trained using normal images does not perform well.

### Future scope

In this era of large models, it can be anticipated that a general large model for registration will soon emerge. With the ability to use human-marked point pairs or corresponding mask areas as registration prompts, this model can be trained on higher quality, broader types, and more extensive image registration datasets, allowing for better generalization.

There remain many areas in which retinal image registration can be explored. With multiple imaging modalities, there is a pressing need for multimodal image registration. To address this issue, translation-based and disentangling representation methods may be new approaches. Interestingly, any pioneers attempting transformer-based retinal registration methods that could lead to even greater accuracy was not observed.

Moreover, data scarcity remains a significant challenge; however, this can be overcome through data generation or transfer learning. For instance, the dataset with image pairs could be supplemented through random translation, rotation, brightness, and contrast enhancement using retinal images from other datasets. When employing transfer learning, endoscopic images from other parts of the human body can be trained or virtual datasets can be manually generated and fine-tuned for retinal image registration.

## Conclusions

This study thoroughly analyzed medical image registration, focusing on its application in retinal imaging. The review compares general medical image registration techniques and their adaptation to retinal imaging, highlights gaps in the current research, and provides advice on avenues for future research. State-of-the-art medical image registration methods were also evaluated and the advantages and disadvantages of each method. Finally, challenges specific to retinal registration were identified and potential opportunities for further advancement discussed.

## Data Availability

All relevant data and material are presented in the main paper.

## Abbreviations

CAD: Computer-aided diagnosis
CNN: Convolutional neural network
GAN: Generative adversarial network
AMD: Age-related macular degeneration
CNV: Choroidal neovascularization
CF: Color fundus photography
FA: Fluorescein angiography
OCT: Optical coherence tomography
OCTA: Optical coherence tomography angiography
(R)MSE: (Root) mean square error
SR: Success rate
DSC: Dice similarity coefficient
DR: Diabetic retinopathy
CC: Cross-correlation
MI: Mutual information
SSD: Sum of squared difference
DDPM: Denoising diffusion probabilistic model
ViT: Vision transformer
SIFT: Scale-invariant feature transform
ORB: Oriented FAST and rotated BRIEF
STL: Spatial transformer layer
i2i: Image-to-image
FCN: Fully convolutional networks
